# Prospective evaluation of the impact of stress, anxiety, and depression on household income among young women with early breast cancer from the Young and Strong trial

**DOI:** 10.1186/s12889-020-09562-z

**Published:** 2020-10-06

**Authors:** Erin E. Cook, Shoshana M. Rosenberg, Kathryn J. Ruddy, William T. Barry, Mary Greaney, Jennifer Ligibel, Kim Sprunck-Harrild, Michelle D. Holmes, Rulla M. Tamimi, Karen M. Emmons, Ann H. Partridge

**Affiliations:** 1 Harvard T.H. Chan School of Public Health, Boston, MA USA; 2 Current affiliation: Analysis Group, Inc., Boston, MA USA; 3 Medical Oncology, Dana-Farber Cancer Institute, 450 Brookline Avenue, Boston, MA 02215 USA; 4 Harvard Medical School, Boston, MA USA; 5 Mayo Clinic, Rochester, MN USA; 6 Division of Biostatistics, Department of Data Sciences, Dana-Farber Cancer Institute, Boston, MA USA; 7 University of Rhode Island, Kingston, RI USA

**Keywords:** Breast cancer, Young adult, Income, Anxiety, Stress, Depression

## Abstract

**Background:**

Young women with breast cancer tend to report lower quality of life and higher levels of stress than older women with breast cancer, and this may have implications for other psychosocial factors including finances. We sought to determine if stress, anxiety, and depression at diagnosis were associated with changes in household income over 12-months in young women with breast cancer in the United States.

**Methods:**

This study was a prospective, longitudinal cohort study comprised of women enrolled in the Young and Strong trial. Of the 467 women aged 18–45 newly diagnosed with early-stage breast cancer enrolled in the Young and Strong trial from 2012 to 2013, 356 (76%) answered income questions. Change in household income from baseline to 12 months was assessed and women were categorized as having lost, gained, maintained the same household income <$100,000, or maintained household income ≥$100,000. Patient-reported stress, anxiety, and depression were assessed close to diagnosis at trial enrollment. Adjusted multinomial logistic regression models were used to compare women who lost, gained, or maintained household income ≥$100,000 to women who maintained the same household income <$100,000.

**Results:**

Although most women maintained household income ≥$100,000 (37.1%) or the same household income <$100,000 (32.3%), 15.4% lost household income and 15.2% gained household income. Stress, anxiety, and depression were not associated with gaining or losing household income compared to women maintaining household incomes <$100,000. Women with household incomes <$50,000 had a higher risk of losing household income compared to women with household incomes ≥$50,000. Women who maintained household incomes ≥$100,000 were less likely to report financial or insurance problems. Among women who lost household income, 56% reported financial problems and 20% reported insurance problems at 12 months.

**Conclusions:**

Baseline stress, anxiety, and depression were not associated with household income changes for young women with breast cancer. However, lower baseline household income was associated with losing household income. Some young survivors encounter financial and insurance problems in the first year after diagnosis, and further support for these women should be considered.

**Trial registration:**

Clinicaltrials.gov, NCT01647607; date registered: July 23, 2012.

## Introduction

While only 7% of breast cancer cases were diagnosed among women under age 40 in the United States in 2017, breast cancer is the most common cancer among adolescent and young adult women ages 15–39 in the United States [1, 2]. Breast cancer is often more aggressive in young women than older women; however, the relative 5-year survival for women ages 35–39 is still high at nearly 90% [2, 3]. Nevertheless, young women with breast cancer tend to report lower quality of life (QOL) and higher levels of stress than older women with breast cancer both at diagnosis and in longer term follow-up [4–7]. This increased distress may impair subsequent psychosocial outcomes including employment, and the burden of employment and financial disturbances after treatment in younger cancer survivors may further impact their subsequent QOL outcomes [4, 5, 7–9]. These financial and employment disturbances can impact both the woman with cancer and her family members [10].

Employment opportunities and financial stability are of particular concern for this younger cohort of women with breast cancer since most women are in the start or prime of their careers and have young families at the time of diagnosis. Financial and social support for cancer patients vary from country to country based on the policies and socio-cultural norms of each country. Additionally, economic conditions and current policy can change. For example, the economic recession in 2008 and the introduction of the Affordable Care Act in 2010 had large impacts on cancer patients’ financial situations. Additionally in the United States, some laws impact patients with cancer, such as the Family and Medical Leave Act (FMLA) which requires employers to allow unpaid leave for certain employees and the Americans With Disabilities Act (ADA) [11] which requires employers to make reasonable accommodations for employees with cancer. However, disruption to employment during this time can be challenging due to employment’s link to financial stability and health insurance, as well as the added stress and anxiety that comes from managing taking time off from work during cancer treatment [12]. Previous research has noted that cancer patients face financial challenges from their care, such as loss of income due to work absences, that can result in reducing spending on other necessities such as food or other bills [13, 14]. These financial burdens, understandably, can result in additional stress and worry for cancer patients [15–17].

However, the impact of stress, anxiety and depression at diagnosis on changes to employment and income levels has not been examined in as much detail in cancer patients. In other populations, some research has explored the association between stress, anxiety and depression with changes in employment. For example, post-traumatic stress disorder and depression have been associated with lower employment [18, 19]. Additionally, a systematic literature review on return to work among breast cancer survivors found that depression and emotional distress were barriers of returning to work [20]. Since a cancer diagnosis is a stressful event that can results in higher levels of stress, depression and/or anxiety, it is of interest to understand if they have any impact on income [21–23]. This study aimed to determine if stress, anxiety, or depression is associated with household income changes for young women with breast cancer over the first 12 months after diagnosis. Household income was used based its association with well-being of individuals and populations as well as its availability and common use by economists [24–26].

## Methods

### Study population

The randomized trial, Young and Strong: An Education and Supportive Care Intervention Study for Young Women with Breast Cancer (NCT01647607), which was led by the Dana- Farber Cancer Institute, provided data for this study [27, 28]. This trial enrolled 467 English-speaking women ages 18–45 with newly diagnosed stage I-III breast cancer. Enrollment occurred from July 2012 to December 2013 at 14 academic and 40 community practices around the United States. Practices were randomized to the Young Women’s Intervention (YWI) or the Physical Activity Intervention (PAI). The YWI contained information about fertility, genetic testing, physical activity, and survivorship. The PAI contained information about physical activity. Participants completed surveys at baseline, 3, 6, and 12 months. Baseline assessment often occurred at the first medical oncology visit for the participants. Details on the trial can be found in previous publications [27, 28].

Since the YWI discussed how breast cancer impacts employment, we examined if the intervention arm impacted change in income and found no statistically significant associations (eTable 1). Thus, we combined the YWI and PAI arms for the rest of this analysis. This study was a prospective, longitudinal cohort study of women who participated in the Young and Strong trial. Women who did not respond to the 12-month survey (*N* = 51), had stage IV disease (*N* = 2), and did not report household income on the baseline and 12-month surveys (*N* = 58) were excluded, leaving an analytic sample of 356 women. Women excluded for missing income information tended to be older and less educated than women included (eTable 2).

### Exposures

The main exposures were baseline measures of stress, anxiety, and depression. Stress was measured using the Perceived Stress Scale (PSS) with categories of low (< 14), moderate (14–26), or high (≥ 27) stress [29]. Anxiety was measured from the Hospital Anxiety and Depression Scale (HADS) anxiety subscale with categories of normal (< 8), borderline anxious (8–10), or anxious (≥ 11) [30, 31]. Depression was measured by the Center for Epidemiological Studies Depression Scale (CES-D) and was a binary variable of no depression (< 16) or depression (≥ 16) [32]. We used the CES-D over the HADS depression subscale due to its better ability to detect major depression [33].

### Outcomes

The primary outcome was change in household income between the baseline and 12-month survey. Household income refers to the combined income of all people living together in a household. Women self-reported their household income from all sources before taxes in a categorical variable (<$5000, $5000–$11,999, $12,000–$15,999, $16,000–$24,999, $25,000–$34,999, $35,000–$49,999, $50,000–$74,999, $75,000–$99,999, and ≥ $100,000). We created a categorical variable for change in household income: losing, gaining, maintaining ≥$100,000, or maintaining same household income <$100,000. We were unable to determine how household incomes changed if women maintained household incomes ≥ $100,000 based on the categories of the household income question (ex: $150,000 to $200,000 or vice versa). Therefore, we separated women who reported the same household income category at baseline and 12-months into maintaining the same household income <$100,000 and maintaining household income ≥ $100,000. We were also unable to determine how household incomes changed if women maintained household incomes <$5000; however, since this total was so low and the category was relatively small we did not separate out these women. Women were categorized as losing or gaining household income, regardless of their household income category, if they reported a different household income category at baseline and 12-months.

Women reported financial, insurance, and working concerns on the 3- and 12-month surveys using a modified CAncer Rehabilitation Evaluation System. Short Form (CARES-SF) questionnaire [34]. For all women we examined employment status and responses to the questions “I have financial problems” and “I have insurance problems.” Employed women were asked: “I have difficulty talking to people who work with me about the cancer,” “I have difficulty asking for time off work for medical treatments,” and “I am worried about being fired.” Unemployed women were asked if they looked for work in the past month. Responses to these questions were categorized as no if respondents reported “not at all” or “a little” and yes if respondents reported “a fair amount,” “much,” or “very much.”

### Statistical analysis

Descriptive statistics, including the number, percentage, and chi-square or Fisher’s exact test (when ≤5 women), were calculated by change in income for demographic, cancer, psychosocial, employment, and financial information. Separate multinomial logistic regression models, with the reference category being same household income <$100,000, analyzed how stress, anxiety, and depression were associated with changes in household income. Models were adjusted for age (continuous), race/ethnicity (Hispanic, Black, other vs. White), marital status (yes vs. no), children (yes vs. no), stage, and baseline household income (≥ $50,000 vs. <$50,000). We performed multiple sensitivity analyses. First, we did not have employment information at baseline, so we added 3-month employment status as a proxy. Second, we created propensity scores and included them as quintiles in the regression models. Lastly, since the survey household income categories were not uniform ranges ($5000–$25,000), we grouped women into even income categories at baseline and 12 months (<$25,000, $25,000–$49,999, $50,000–$74,999, $75,000–$99,999, and ≥ $100,000). From these categories we looked at women who lost, gained, maintained the same income <$100,000, and maintained incomes ≥ $100,000.

Results were considered statistically significant if *p* < 0.05. SAS 9.4 was used for the cluster randomized analyses and StataIC 14 was used for the remaining analyses [35, 36]. The study was approved by the Dana-Farber Cancer Institute Institutional Review Board, which oversaw most of the study sites; however, some sites maintained their own institutional review. Written informed consent was obtained from all participants prior to study enrollment.

## Results

Over 12 months, 37% of women maintained household incomes ≥ $100,000, 32% maintained the same household income <$100,000, 15% gained household income, and 15% lost household income (Table 1). Women in the income change categories were similar to each other in terms of demographic, cancer characteristics, and psychosocial measures. However, women maintaining incomes ≥ $100,000 were more likely than women in the other household income categories to be more educated, married, and have stage I disease. They were less likely to have chemotherapy and depression. The proportion of women experiencing high stress ranged from 3.1% (women maintaining ≥ $100,000) to 13.2% (women losing income). Anxiety ranged from 30.7% of women with the same income <$100,000 to 42.4% of women gaining income. Depression ranged from 31.5% of women maintaining ≥ $100,000 to 54.0% of women losing income. Among women who lost or gained household income, the estimated dollar amount of change in household income was similar (eTable 3).

**Table 1:** Participant characteristics at baseline survey by change in household income for women in the Young and Strong trial in 2012–2013

	Change in Household Income				
	Maintain ≥ $100,000 (***N*** = 132)	Same < $100,000 (***N*** = 115)	Gained (***N*** = 54)	Lost (***N*** = 55)	
	N. (%)	N. (%)	N. (%)	N. (%)	*p* value
**Demographics**					
Age					0.16
< 35	18 (13.6)	30 (26.1)	13 (24.1)	15 (27.3)	
35–39	38 (28.8)	23 (20.0)	15 (27.8)	11 (20.0)	
40–45	76 (57.6)	62 (53.9)	26 (48.2)	29 (52.7)	
Education					< 0.0001^a^
≤ High School	1 (0.8)	13 (11.3)	11 (20.4)	10 (18.2)	
≥ Some College	131 (99.2)	102 (88.7)	43 (79.6)	45 (81.8)	
Married					< 0.0001
Yes	122 (92.4)	82 (71.3)	43 (79.6)	41 (74.6)	
No	10 (7.6)	33 (28.7)	11 (20.4)	14 (25.5)	
Children					0.07
Yes	107 (81.1)	78 (67.8)	36 (67.7)	40 (74.1)	
No	25 (18.9)	37 (32.2)	18 (33.3)	14 (25.9)	
Race/Ethnicity					0.31
Non-Hispanic, White	108 (81.8)	85 (74.6)	38 (70.4)	41 (74.6)	
Hispanic, Black, Other	24 (18.2)	29 (25.4)	16 (29.6)	14 (25.5)	
Region					0.19
Northeast	38 (28.8)	30 (26.1)	11 (20.4)	12 (21.8)	
South/Southeast	22 (16.7)	34 (29.6)	18 (33.3)	18 (32.7)	
Midwest	46 (34.9)	38 (33.0)	15 (27.8)	18 (32.7)	
West	26 (19.7)	13 (11.3)	10 (18.5)	7 (12.7)	
Change in Employment					0.23^a^
None-Employed	85 (68.0)	66 (62.3)	26 (54.2)	33 (63.5)	
None-Unemployed	20 (16.0)	20 (18.9)	11 (22.9)	15 (28.9)	
Lost	1 (0.8)	0 (0.0)	1 (2.1)	0 (0.0)	
Gained	19 (15.2)	20 (18.9)	10 (20.8)	4 (7.7)	
Household Income					< 0.0001
< $50,000	0 (0.0)	32 (27.8)	37 (68.5)	23 (41.8)	
$50,000–$99,999	0 (0.0)	83 (72.2)	17 (31.5)	21 (38.2)	
≥ $100,000	132 (100.0)	0 (0.0)	0 (0.0)	11 (20.0)	
**Cancer**					
Stage					0.01
I	62 (47.0)	39 (33.9)	12 (22.2)	14 (25.5)	
II	54 (40.9)	61 (53.0)	31 (57.4)	28 (50.9)	
III	16 (12.1)	15 (13.0)	11 (20.4)	13 (23.6)	
Estrogen Receptor					0.19
Positive	107 (81.1)	82 (71.3)	37 (68.5)	40 (72.7)	
Negative	25 (18.9)	33 (28.7)	17 (31.5)	15 (27.3)	
Progesterone Receptor					0.39
Positive	99 (75.0)	79 (68.7)	36 (66.7)	35 (63.6)	
Negative	33 (25.0)	36 (31.3)	18 (33.3)	20 (36.4)	
HER2					0.91
Positive	30 (23.1)	26 (22.8)	12 (22.2)	15 (27.3)	
Negative	100 (76.9)	88 (77.2)	42 (77.8)	40 (72.7)	
**Baseline Cancer Treatment**					
Chemotherapy					0.01
Yes/Planned	96 (73.3)	97 (85.8)	48 (88.9)	48 (88.9)	
No	35 (26.7)	16 (14.2)	6 (11.1)	6 (11.1)	
Radiation					0.05
Yes/Planned	69 (61.6)	59 (64.8)	39 (83.0)	31 (72.1)	
No	43 (38.4)	32 (35.2)	8 (17.0)	12 (27.9)	
Endocrine Therapy					0.53
Yes/Planned	97 (78.9)	78 (72.2)	34 (69.4)	37 (74.0)	
No	26 (21.1)	30 (27.8)	15 (30.6)	13 (26.0)	
**Baseline Psychosocial Measures**					
Stress					0.15^a^
Low	52 (40.3)	42 (37.8)	16 (30.2)	14 (26.4)	
Moderate	73 (56.6)	61 (55.0)	32 (60.4)	32 (60.4)	
High	4 (3.1)	8 (7.2)	5 (9.4)	7 (13.2)	
Anxiety					0.73
Normal	55 (42.0)	50 (43.9)	19 (35.9)	20 (37.7)	
Borderline	31 (23.7)	29 (25.4)	11 (20.8)	16 (30.2)	
Anxiety	45 (34.4)	35 (30.7)	23 (42.4)	17 (32.1)	
Depression					0.03
No	85 (68.6)	63 (58.3)	27 (54.0)	23 (46.0)	
Yes	39 (31.5)	45 (41.7)	23 (46.6)	27 (54.0)	

(^a^)Fisher’s exact test; N=Number; missing: 1 children, 1 race/ethnicity, 25 employment, 3 HER2, 4 chemotherapy, 63 radiation, 26 endocrine therapy, 10 stress, 5 anxiety, 24 depression; 100% had/planned surgery; p -values were calculated using chi-square tests or Fisher’s exact tests (when ≤5 women)

Psychosocial measures, including stress, anxiety and depression, were not associated with losing, gaining, or maintaining household incomes ≥ $100,000 compared to maintaining the same household income <$100,000 (Table 2). Specifically, losing household income was not associated with high stress (Risk ratio, RR = 2.42, 95% confidence internal [CI] 0.72–8.08), anxiety (RR = 1.12, 95% CI 0.50–2.50), or depression (RR = 1.41, 95% CI 0.70–2.85) at baseline. Women reporting baseline household income <$50,000 were 2.23 times higher risk of losing income compared to those reporting household incomes ≥ $50,000 (95% CI 1.04–4.78). Women with stage III disease had a 2.68 times higher risk of losing income than stage I disease with borderline statistical significance (95% CI 0.98–7.37). Women with baseline household incomes <$50,000 compared to ≥ $50,000 were more likely to gain income (RR = 8.47, 95% CI 3.87–18.81). Married women were more likely to gain or maintain household income ≥ $100,000 than unmarried women (RR = 3.76, 95% CI 1.50–9.41; RR = 2.56, 95% CI 1.08–6.06; respectively). Similar results were seen when the models were adjusted using propensity score quintiles (eTable 4), adjusted for 3-month employment, and when we used the change in household income variable from the uniform income categories (data not shown).

**Table 2:** Adjusted multinomial logistic regression analysis of stress, anxiety and depression’s impact on change in household income among young women with breast cancer

	Lost vs. Same < $100,000		Gained vs. Same < $100,000		Maintain ≥ $100,000 vs. Same < $100,000	
	RR (95% CI)	*p* value	RR (95% CI)	*p* value	RR (95% CI)	*p* value
**Stress**						
Low	Ref.		Ref.		Ref.	
Moderate	1.35 (0.63–2.91)	0.44	0.96 (0.43–2.14)	0.91	0.97 (0.55–1.74)	0.93
High	2.42 (0.72–8.08)	0.15	1.47 (0.38–5.72)	0.58	0.43 (0.11–1.65)	0.22
**Anxiety**						
Normal	Ref.		Ref.		Ref.	
Borderline	1.16 (0.50–2.67)	0.73	0.88 (0.34–2.28)	0.80	1.05 (0.52–2.11)	0.89
Anxiety	1.12 (0.50–2.50)	0.79	1.50 (0.65–3.47)	0.34	1.26 (0.66–2.40)	0.48
**Depression**						
No	Ref.		Ref.		Ref.	
Yes	1.41 (0.70–2.85)	0.34	0.90 (0.42–1.93)	0.78	0.73 (0.41–1.31)	0.29
**Covariates Only**						
Age	0.99 (0.93–1.05)	0.71	1.01 (0.94–1.08)	0.86	1.04 (0.98–1.10)	0.15
Cancer Stage						
I	Ref.		Ref.		Ref.	
II	1.36 (0.62–2.98)	0.57	1.64 (0.70–3.84)	0.26	0.64 (0.35–1.14)	0.13
III	2.68 (0.98–7.37)	0.06	2.72 (0.88–8.37)	0.08	0.75 (0.31–1.83)	0.53
Married						
No	Ref.		Ref.		Ref.	
Yes	1.69 (0.72–3.92)	0.23	3.76 (1.50–9.41)	0.005	2.56 (1.08–6.06)	0.03
Children						
No	Ref.		Ref.		Ref.	
Yes	1.21 (0.53–2.76)	0.65	0.71 (0.30–1.65)	0.42	1.49 (0.76–2.92)	0.25
Race/Ethnicity						
Non-Hispanic, White	Ref.		Ref.		Ref.	
Hispanic, Black, other	1.26 (0.57–2.76)	0.57	1.32 (0.59–3.06)	0.49	1.10 (0.56–2.19)	0.78
Baseline Income						
≥ $50,000	Ref.		Ref.			
< $50,000	2.23 (1.04–4.78)	0.04	8.47 (3.87–18.81)	< 0.0001		

Separate models were run for stress, anxiety, depression and covariates only; models were adjusted for age, marriage, children, stage, race/ethnicity, and baseline income; *CI* Confidence interval; *RR* Risk ratio

Women maintaining incomes ≥ $100,000 reported financial and insurance problems less frequently than women in the other household income change categories (Table 3). Of women maintaining incomes ≥ $100,000, 5.3% at 3 months and 7.6% at 12 months reported financial problems, compared to 49.1% at 3 months and 56.4% at 12 months of women losing income (3 and 12-month *p* < 0.0001). Women maintaining incomes ≥ $100,000 (3 month = 3.0%, 12 month = 5.3%) and women maintaining the same income <$100,000 (3 month = 7.8%, 12 month = 7.0%) reported insurance problems less frequently than women who gained (3 month = 22.2%, 12 month = 11.1%) or lost income (3 month = 25.5%, 12 month = 20.0%) (3 month: *p* < 0.0001, 12 month: *p* = 0.02). Overall, approximately 60% of women were working at 3 months and 79% were working at 12 months.

**Table 3:** Measures of work, financial, and insurance worries by change in household income among young women with breast cancer in the Young and Strong trial from 2012 to 2013

	3 Months					12 Months				
	Maintain ≥ $100,000	Same < $100,000	Gained	Lost		Maintain ≥ $100,000	Same < $100,000	Gained	Lost	
	N. (%)	N. (%)	N. (%)	N. (%)	*p* value	N. (%)	N. (%)	N. (%)	N. (%)	*p* value
**All Women**										
Financial Problems					< 0.0001					< 0.0001
No	119 (90.2)	77 (67.0)	31 (57.4)	25 (45.5)		120 (90.9)	75 (65.2)	34 (63.0)	23 (41.8)	
Yes	7 (5.3)	31 (27.0)	17 (31.5)	27 (49.1)		10 (7.6)	39 (33.9)	20 (37.0)	31 (56.4)	
Missing	6 (4.6)	7 (6.1)	6 (11.1)	3 (5.5)		2 (1.5)	1 (0.9)	0 (0.0)	1 (1.8)	
Insurance Problems					< 0.0001					0.02
No	122 (92.4)	99 (86.1)	36 (66.7)	37 (67.3)		124 (93.9)	106 (92.2)	48 (88.9)	42 (76.4)	
Yes	4 (3.0)	9 (7.8)	12 (22.2)	14 (25.5)		7 (5.3)	8 (7.0)	6 (11.1)	11 (20.0)	
Missing	6 (4.6)	7 (6.1)	6 (11.1)	4 (7.3)		1 (0.8)	1 (0.9)	0 (0.0)	2 (3.6)	
Currently Working					0.50					0.37
No	40 (30.3)	41 (35.7)	21 (38.9)	19 (34.6)		22 (16.7)	22 (19.1)	13 (24.1)	16 (29.1)	
Yes	86 (65.2)	66 (57.4)	27 (50.0)	33 (60.0)		109 (82.6)	92 (80.0)	41 (75.9)	38 (69.1)	
Missing	6 (4.6)	8 (7.0)	6 (11.1)	3 (5.5)		1 (0.8)	1 (0.9)	0 (0.0)	1 (1.8)	
**Employed Women**										
Difficulty Talking to Coworkers					0.61					0.76
No	78 (90.7)	58 (87.9)	22 (81.5)	29 (87.9)		97 (89.0)	84 (91.3)	35 (85.4)	34 (89.5)	
Yes	8 (9.3)	8 (12.1)	5 (18.5)	4 (12.1)		12 (11.0)	8 (8.7)	6 (14.6)	4 (10.5)	
Difficulty Asking for Time Off					0.37					0.78
No	78 (90.7)	55 (83.3)	23 (85.2)	31 (93.9)		98 (89.9)	81 (88.0)	37 (90.2)	32 (84.2)	
Yes	8 (9.3)	11 (16.7)	4 (14.8)	2 (6.1)		11 (10.1)	11 (12.0)	4 (9.8)	6 (15.8)	
Worried about being Fired					0.08					0.001
No	82 (95.4)	57 (86.4)	24 (88.9)	27 (81.8)		106 (97.3)	83 (90.2)	41 (100)	30 (79.0)	
Yes	4 (4.7)	9 (13.6)	3 (11.1)	6 (18.2)		3 (2.8)	9 (9.8)	0 (0.0)	8 (21.1)	
**Unemployed Women**										
Looked for Work in the Past Month					0.08					0.69
No	40 (100.0)	39 (97.5)	21 (100)	17 (89.5)		21 (95.5)	20 (90.9)	10 (83.3)	14 (87.5)	
Yes	0 (0.0)	1 (2.5)	0 (0.0)	2 (10.5)		1 (4.6)	2 (9.1)	2 (16.7)	2 (12.5)	

All *p* from Fisher’s exact tests; N=Number; missing: look for work: 1 at 3 months, 1 at 12 months

The proportion of employed women was similar among household income change categories (Table 3). Two women lost employment between the 3 and 12-month surveys, while 53 gained employment, 210 remained employed, and 66 remained unemployed (Table 1). Among employed women, worries about discussing cancer with coworkers or taking time off work were similar across household income change categories. At 3 months, the percentage worried about being fired was highest among women with the same income <$100,000 (13.6%) and women losing income (18.2%). At 12 months, the percentage worried about being fired varied by income change (0% gaining income to 21% losing income, *p* = 0.001). A small proportion of unemployed women reported looking for work in the past month.

## Discussion

In this study of young women with breast cancer, stress, anxiety and depression following diagnosis were not associated with changes in household income over the subsequent year. However, our finding that lower household income was associated with losing household income suggests that these lower income women may be more vulnerable to income loss, and clinicians caring for these women may want to proactively offer available resources to address financial needs.

Most QOL literature examines how the financial burden of cancer influences QOL, rather than looking at the impact that psychosocial factors can have on finances [7, 37, 38]. However, the amount of distress felt at diagnosis may impact how women balance treatment with work obligations, which in turn may affect their personal income. This study found that stress, anxiety, and depression were not associated with losing or gaining household income, suggesting that the presence of these psychosocial factors may not create a burden large enough to impact household income. A review found that psychosocial factors such as depression and distress made it hard for women with breast cancer to return to work [20]. Additionally, a study using the Surveillance, Epidemiology, and End Results (SEER) database found that 27% of women reported decreasing work hours due to cancer-related health issues [39]. However, in this study only two women lost employment, while 53 women gained employment between 3 and 12 months. Of note, other studies have reported that a higher proportion of women stop working after a breast cancer diagnosis [5, 38]. The difference in our findings may be due to the lack of baseline employment and student status data. However, we examined medical records for unemployed women and noted few changes in employment after diagnosis. Furthermore, not returning to work may not be directly linked to changes in household income if other people in the household, such as her partner or parent, compensate for any lost income women with cancer may personally experience. Since this study only examined changes in household income, the measure of household income in this study is likely a combination of the woman’s income as well as her partner’s income as 81% of the study sample was married.

In our study, 15% of women reported losing household income; however, not many women appeared to lose their job and baseline stress, anxiety and depression were not associated with loss of household income. Without large amounts of unemployment for women with breast cancer, lost household income may have been the result of reduced hours for employed women; however, data on hours worked was not collected. Additionally, we do not know if women who remained employed did so because they enjoyed their job or if they felt locked into their job and were afraid to change positions due to worries about insurance or finances. The loss of household income could also be related to income loss for her partner or other member of the household. Partners of women with breast cancer also face psychosocial and economic distress [40–42]. One prior study found that 32% of partners reduced working hours to help a partner though treatment and 32% reported a worse financial status [10]. Another found that 5% of caregivers had to quit their job [43]. While our study did not specifically look at the partner or other members of the household, and there was no way to disaggregate the measure of household income, it’s possible that part of the financial loss seen among some participants may have resulted from the impact on the partners’ ability to work. However, this study found that married women were more likely to gain income, so the impact of the partner’s income is hard to disentangle from the patient’s income in this study. Further research examining the impact of cancer diagnosis and treatment on the patient as well as the partner/household could help elucidate this relationship.

While stress, anxiety and depression were not associated with changes in household income, this study found that losing household income was associated with lower baseline household incomes and possibly more advanced disease. Similar findings were found in a SEER study where women with incomes <$50,000 had 1.77 times higher odds of a worsening financial status due to breast cancer than women with incomes ≥ $50,000 [39]. Women with high household incomes may have more resources available that lessen the risk of losing household income and may ease the financial toxicity associated with cancer treatment. In our study, women who maintained incomes ≥$100,000 were less likely to report financial problems after 12 months than women in the other income categories (8% vs 34% that maintained income <$100,00, 37% that gained income and 56% that lost income). Additionally, women with later stage disease, regardless of income, may find treatments particularly disruptive which may result in reduced work hours. However, similar to our results, the SEER study found that stage III vs. stage I disease was borderline associated with worsening financial statuses (odds ratio = 1.92, *p* = 0.06) [39].

We also found that women with lower incomes were more likely to gain income. Some women with lower incomes and/or their partners may have been students and their income may have increased upon graduation in this young population. Lower income women may have gained income if they increased work hours to cover costs or gain health insurance coverage. In our study, 22% of women gaining income reported insurance problems at 3 months. The SEER study found that 7% of women increased work hours to cover cancer-related medical expenses [39]. Interestingly, the group of women changing income levels, be it lost or gained, tended to have lower incomes. This may highlight that poorer women experience more volatility around cancer treatment, and more research looking at the reasons for these observations is needed.

### Study limitations

First, the relatively small number of women whose incomes changed limited the power of the analysis and the number of covariates we could adjust for. We performed a propensity score adjusted analysis adjusting for additional covariates and obtained similar results. Second, we only had categorical income information which limited our ability to track all income changes. We were unable to look at changes for women maintaining ≥ $100,000, so our results may not be generalizable to those women. Also, our main outcome was change in household income over 12 months, but we did not collect information about the partner’s employment or any longer term impacts. A final limitation with the income measure was that the information on household income was self-reported by participants and women may have interpreted what should be included in the measure of household income differently. For example, young women in college may or may not choose to report income and support from her parents in her understanding of her own household income.

Third, we were unable to adjust for potentially important confounders including social support and insurance status in the regression models of stress, anxiety and depression’s impact on change in household income. If we had this information, we would expect the results from these regression models to be lower in magnitude than what we saw, due to the inverse relationship between these variables and psychosocial measures as well as income change. Fourth, because the trial occurred from 2012 to 2014, the financial crisis in 2008 and the Affordable Care Act in 2010 may have impacted the insurance and income stability of participants. Fifth, all information was self-reported, so there could be misclassification of income and psychosocial measures. Nonetheless, we believe that any misclassification was likely non-differential. Lastly, women who participated in this trial may be different from the larger population of young breast cancer patients. While this is a national sample recruited from both community and academic sites, women in this study may be more health conscious, and have better financial or personal resources.

## Conclusions

Stress, anxiety and depression were not associated with changes in household income in this study. However, young women with breast cancer with lower household incomes had a higher risk of losing household income than women with higher household incomes. This group of women may need more support during treatment and early survivorship from healthcare providers. Further research to understand the mechanism of income loss for young women with breast cancer, as well as research to understand interventions to support women at risk of losing income will be important to improving the care of young women with breast cancer. Additionally, research to understand the broader burden of financial toxicity for young women with breast cancer will help providers understand which patients could benefit from more support.

## Supplementary information

**Additional file 1: eTable 1**: Analysis by randomization arm (YWI vs PAI). **eTable 2**: Comparison of women included to women excluded. **eTable 3**: Amount of change in household income by income at baseline. **eTable 4**: Comparison of multinomial logistic regression model to the propensity score adjusted logistic regression model for the impact of stress, depression, and anxiety at baseline on changing household income vs maintaining same household income <$100,000.

## Data Availability

The datasets generated during and/or analyzed during the current study are available from the corresponding author on reasonable request.

## Abbreviations

ADA: Americans with disabilities act
CARES-SF: Cancer rehabilitation evaluation system. short form
CES-D: Center for epidemiologic studies depression scale
CI: Confidence interval
FMLA: Family and medical leave act
HADS: Hospital anxiety and depression scale
PAI: Physical activity intervention
PSS: Perceived stress scale
QOL: Quality of life
RR: Risk ratio
SEER: Surveillance, epidemiology, and end results
YWI: Young women’s intervention
