# Supplementary material for: Prospective evaluation of the impact of stress, anxiety, and depression on household income among young women with early breast cancer from the Young and Strong trial
Source: BMC Public Health. 2020 Oct 6;20:1514. doi: 10.1186/s12889-020-09562-z (PMC7541223; doi:10.1186/s12889-020-09562-z)
Supplement: Supplementary file 1 — Additional file 1: eTable 1: Analysis by randomization arm (YWI vs PAI). eTable 2: Comparison of women included to women excluded. eTable 3: Amount of change in household income by income at baseline. eTable 4: Comparison of multinomial logistic regression model to the propensity score adjusted logistic regression model for the impact of stress, depression, and anxiety at baseline on changing household income vs maintaining same household income <$100,000. [file 12889_2020_9562_MOESM1_ESM.docx]

**SUPPLEMENTAL MATERIAL**

**Prospective Evaluation of the Impact of Stress, Anxiety, and Depression on Household Income among Young Women with Early Breast Cancer from the Young and Strong Trial**

Erin E. Cook, MPH, ScD; Shoshana M Rosenberg, ScD, MPH; Kathryn J Ruddy, MD, MPH; William T. Barry, PhD; Mary Greaney, PhD; Jennifer Ligibel, MD; Kim Sprunck-Harrild, MSW, MPH; Michelle D. Holmes, MPH, MD, DrPH; Rulla M. Tamimi, ScD; Karen M. Emmons, PhD; Ann H. Partridge, MD, MPH

**Corresponding author**:

Ann Partridge, MD, MPH

Dana-Farber Cancer Institute

450 Brookline Avenue

Boston, MA 02215

Tel: 617-632-3800

Fax: 617-632-1930

[Ann_Partridge@dfci.harvard.edu](mailto:Ann_Partridge@dfci.harvard.edu)

Supplemental tables: 4

**eTable 1: Analysis by randomization arm (YWI vs PAI)**

|  | **Odds Ratio** | **(95% CI)** | **p value** |
| --- | --- | --- | --- |
| **Household Income** |  |  |  |
| Income Lost (vs gain or same) | 0.79 | (0.44-1.41) | 0.43 |
| Income Gained (vs lost or same) | 1.52 | (0.85-2.77) | 0.16 |
| Income Changed (vs same) | 1.12 | (0.71-1.77) | 0.62 |
| Income Lost vs no change | 0.85 | (0.47-1.53) | 0.59 |
| Income Gained vs no change | 1.49 | (0.82-2.72) | 0.19 |

Each row is a separate regression model, generalized estimating equations were used to account for within-cluster correlations, an exchangeable correlation structure was used, YWI = young women’s intervention, PAI = physical activity intervention, CI = confidence interval

**eTable 2: Comparison of women included to women excluded**

|  | **Income information known (N=356)**  N. (%) | **Income information not complete (N=58)**  N. (%) | p value | |
| --- | --- | --- | --- | --- |
| **Demographics** |  |  |  | |
| Age |  |  | 0.04^a^ | |
| <35 | 76 (21.4) | 5 (8.6) |  | |
| 35-39 | 87 (24.4) | 20 (34.5) |  | |
| 40-45 | 193 (54.2) | 33 (56.9) |  | |
| Education |  |  | 0.02 | |
| ≤High School | 35 (9.8) | 12 (20.7) |  | |
| ≥Some College | 321 (90.2) | 46 (79.3) |  | |
| Married |  |  | 0.52 | |
| Yes | 288 (80.9) | 49 (84.5) |  | |
| No | 68 (19.1) | 9 (15.5) |  | |
| Children |  |  | 0.02 | |
| Yes | 261 (73.5) | 51 (87.9) |  | |
| No | 94 (26.5) | 7 (12.1) |  | |
| Race/Ethnicity |  |  | 0.85 | |
| Non-Hispanic, White | 272 (76.6) | 43 (75.4) |  | |
| Minority | 83 (23.4) | 14 (24.6) |  | |
| Region |  |  | 0.43 | |
| Northeast | 91 (25.6) | 14 (24.1) |  | |
| South and Southeast | 92 (25.8) | 13 (22.4) |  | |
| Midwest | 117 (32.9) | 25 (43.1) |  | |
| West | 56 (15.7) | 6 (10.3) |  | |
| Change in Employment |  |  | 0.002^a^ | |
| None–Employed | 210 (63.4) | 23 (41.8) |  | |
| None–Unemployed | 66 (19.9) | 19 (34.6) |  | |
| Lost job | 2 (0.6) | 3 (5.5) |  | |
| Gained job | 53 (16.0) | 10 (18.2) |  | |
| Household Income |  |  | <0.0001^a^ | |
| <$50,000 | 92 (25.8) | 9 (15.5) |  | |
| $50,000-$99,999 | 121 (24.0) | 5 (8.6) |  | |
| ≥$100,000 | 143 (40.2) | 12 (20.7) |  | |
| Missing | 0 (0.0) | 32 (55.2) |  | |
| **Cancer and Baseline Cancer Treatment** | |  |  | |
| Stage |  |  | 0.52 | |
| I | 127 (35.7) | 20 (34.5) |  | |
| II | 174 (48.9) | 32 (55.2) |  | |
| III | 55 (15.5) | 6 (10.3) |  | |
| Estrogen Receptor |  |  | 0.85 | |
| Positive | 266 (74.7) | 44 (75.9) |  | |
| Negative | 90 (25.3) | 14 (24.1) |  | |
| Progesterone Receptor |  |  | 0.23 | |
| Positive | 249 (69.9) | 45 (77.6) |  | |
| Negative | 107 (30.1) | 13 (22.4) |  | |
| Her2 |  |  | 0.50 | |
| Positive | 83 (23.5) | 16 (27.6) |  | |
| Negative | 270 (76.5) | 42 (72.4) |  | |
|  | **Income information known (N=356)**  N. (%) | **Income information not complete (N=58)**  N. (%) | p value |  |
| Chemotherapy |  |  | 0.44 |  |
| Yes/Planned | 289 (82.1) | 50 (86.2) |  |  |
| No | 63 (17.9) | 8 (13.8) |  |  |
| Radiation |  |  | 0.55 |  |
| Yes/Planned | 198 (67.6) | 31 (63.3) |  |  |
| No | 95 (32.4) | 18 (36.7) |  |  |
| Endocrine Therapy |  |  | 0.46 |  |
| Yes/Planned | 246 (74.6) | 42 (79.3) |  |  |
| No | 84 (25.5) | 11 (20.8) |  |  |
| **Baseline Psychosocial Measures** | |  |  |  |
| Stress |  |  | 0.84^a^ |  |
| Low | 124 (35.8) | 21 (38.9) |  |  |
| Moderate | 198 (57.2) | 29 (53.7) |  |  |
| High | 24 (6.9) | 4 (7.4) |  |  |
| Anxiety |  |  | 0.88 |  |
| Normal | 144 (41.0) | 25 (44.6) |  |  |
| Borderline | 87 (24.8) | 13 (23.2) |  |  |
| Anxiety | 120 (34.2) | 18 (32.1) |  |  |
| Depression |  |  | 0.60 |  |
| No | 198 (59.6) | 33 (63.5) |  |  |
| Yes | 134 (40.4) | 19 (36.5) |  |  |

(^a^)Fisher’s exact test; N=Number; unknown values not shown: 1 child, 2 race/ethnicity, 28 employment, 3 HER2, 4 chemotherapy, 72 radiation, 31 endocrine therapy, 14 stress, 7 anxiety, 30 depression; 100% of women had/planned surgery

**eTable 3: Amount of change in household income by income at baseline**

|  | **Change in Household Income** | |
| --- | --- | --- |
|  | **Loss (N=55)** | **Gained (N=54)** |
| Amount Gained or Loss | N. (%) | N. (%) |
| All Incomes |  |  |
| $20,001 or more | 27 (49.1) | 27 (50.0) |
| $10,001 to $20,000 | 17 (30.9) | 19 (35.2) |
| $5,001 to $10,000 | 11 (20.0) | 8 (14.8) |
| Incomes <$25,000 at baseline |  |  |
| $20,001 or more | - | 6 (35.3) |
| $10,001 to $20,000 | 2 (2.0.0) | 3 (17.7) |
| $5,001 to $10,000 | 8 (80.0) | 8 (47.1) |
| Incomes $25,000-$49,999 at baseline |  |  |
| $20,001 or more | 4 (30.8) | 4 (20.0) |
| $10,001 to $20,000 | 6 (46.2) | 16 (80.0) |
| $5,001 to $10,000 | 3 (23.1) | - |
| Incomes $50,000-$74,999 at baseline |  |  |
| $20,001 or more | 1 (10.0) | 10 (100.0) |
| $10,001 to $20,000 | 9 (90.0) | - |
| $5,001 to $10,000 | - | - |
| Incomes $75,000-$99,999 at baseline |  |  |
| $20,001 or more | 11 (100.0) | 7 (100.0) |
| $10,001 to $20,000 | - | - |
| $5,001 to $10,000 | - | - |
| Incomes ≥ $100,000 at baseline |  |  |
| $20,001 or more | 11 (100.0) | - |
| $10,001 to $20,000 | - | - |
| $5,001 to $10,000 | - | - |

To calculate the amount in the change in income the average of each income category was chosen for the value used in the subtraction of (income at 12 months – income at baseline), for incomes $100,000+ the value $125,000 was assigned, N = number, values not shown were unable to be determined from the dataset due to the categories used

**eTable 4: Comparison of multinomial logistic regression model to the propensity score adjusted logistic regression model for the impact of stress, depression, and anxiety at baseline on changing household income vs maintaining same household income <$100,000**

|  | **Multinomial Logistic Regression** | | | | **Propensity Score Adjusted Logistic Regression** | | | |
| --- | --- | --- | --- | --- | --- | --- | --- | --- |
|  | **Lose Income** | | **Gain Income** | | **Lose Income** | | **Gain Income** | |
|  | RR (95% CI) | p value | RR (95% CI) | p value | RR (95% CI) | p value | RR (95% CI) | p value |
| Stress |  |  |  |  |  |  |  |  |
| Low | Ref. |  | Ref. |  | Ref. |  | Ref. |  |
| Moderate or High | 1.48 (0.70-3.11) | 0.30 | 1.02 (0.46-2.23) | 0.97 | 1.45 (0.69-3.05) | 0.33 | 1.15 (0.56-2.38) | 0.70 |
| Depression |  |  |  |  |  |  |  |  |
| No | Ref. |  | Ref. |  | Ref. |  | Ref. |  |
| Yes | 1.41 (0.70-2.85) | 0.34 | 0.90 (0.42-1.93) | 0.78 | 1.43 (0.70-2.99) | 0.32 | 0.91 (0.44-1.87) | 0.80 |
| Anxiety |  |  |  |  |  |  |  |  |
| Normal | Ref. |  | Ref. |  | Ref. |  | Ref. |  |
| Borderline or Anxious | 1.14 (0.57-2.28) | 0.71 | 1.21 (0.57-2.56) | 0.62 | 1.20 (0.60-2.41) | 0.60 | 1.36 (0.68-2.73) | 0.38 |

Separate regressions were run for stress, depression and anxiety (each row); the reference category for the multinomial logistic regression is same income <$100,000; the multinomial logistic regression was adjusted for age (continuous), cancer stage, marital status, children, race/ethnicity, and baseline income; the propensity score was created by using a logistic regression to predict each dichotomized exposure status separately, the propensity score model included the variables age (categorical), marital status, race/ethnicity, children, income at baseline, cancer stage, chemotherapy at baseline, and type of medical practice (academic vs. community), the propensity score was split into quintiles and added as a covariate to the multinomial logistic regression; each row is a separate regression model; RR= Risk Ratio; CI = Confidence Interval
